# TLR7 Modulated T Cell Response in the Mesenteric Lymph Node of *Schistosoma japonicum*-Infected C57BL/6 Mice

**DOI:** 10.1155/2019/2691808

**Published:** 2019-12-22

**Authors:** Jiale Qu, Xiuxue Yu, Chenxi Jin, Yuanfa Feng, Shihao Xie, Hongyan Xie, Quan Yang, Yanwei Qi, Huaina Qiu, Hongyuan Chen, Jianbing Mu, Yi Zhou, Jun Huang

**Affiliations:** ^1^The Second Affiliated Hospital, Guangdong Provincial Key Laboratory of Allergy & Clinical Immunology, The State Key Laboratory of Respiratory Disease, Guangzhou Medical University, Guangzhou 510260, China; ^2^Guangdong Pharmaceutical University, Guangzhou 510006, China; ^3^Laboratory of Malaria and Vector Research, National Institute of Allergy and Infectious Diseases, National Institutes of Health, Bethesda, Maryland, USA; ^4^College of Pharmacy, Guangzhou Medical University, 510182 Guangzhou, China; ^5^Key Laboratory of Tropical Diseases Control (Sun Yat-sen University), Ministry of Education, Guangzhou, Guangdong 510080, China

## Abstract

Toll-like receptors (TLRs) play an important role in regulating immune responses during pathogen infection. However, roles of TLRs on T cells reside in the mesenteric lymph node (MLN) were not be fully elucidated in the course of *S. japonicum* infection. In this study, T lymphocytes from the mesenteric lymph node (MLN) of *S. japonicum*-infected mice were isolated and the expression and roles of TLR2, TLR3, TLR4, and TLR7 on both CD4^+^ and CD8^+^ T cells were compared. We found that the expression of TLR7 was increased in the MLN cells of *S. japonicum*-infected mice, particularly in CD4^+^ and CD8^+^ T cells (*P* < 0.05). R848, a TLR7 agonist, could enhance the production of IFN-*γ* from MLN T cells of infected mice (*P* < 0.05), especially in CD8^+^ T cells (*P* < 0.01). In TLR7 gene knockedout (KO) mice, the *S. japonicum* infection caused a significant decrease (*P* < 0.05) of the expression of CD25 and CD69, as well as the production of IFN-*γ* and IL-4 inducted by PMA plus ionomycin on both CD4^+^ and CD8^+^ T cells. Furthermore, the decreased level of IFN-*γ* and IL-4 in the supernatants of SEA- or SWA-stimulated mesenteric lymphocytes was detected (*P* < 0.05). Our results indicated that *S. japonicum* infection could induce the TLR7 expression on T cells in the MLN of C57BL/6 mice, and TLR7 mediates T cell response in the early phase of infection.

## 1. Introduction

Schistosomiasis is a chronic, parasitic disease caused by blood flukes with significant morbidity and mortality, especially in vertebrates, including humans [1]. Immunopathological studies have shown that schistosomiasis results predominantly from the evoked host immune response to schistosome eggs and the granulomatous reaction [2]. After infection, schistosomula and its eggs migrate through a variety of tissues, such as the skin, lung [3], liver [4, 5], and intestinal and vesical mucosa [6]. Schistosoma eggs must migrate from the mesenteric vessels, across the intestinal wall and into the feces. A vast proportion of eggs fail to leave their definite host, instead becoming lodged within intestinal or hepatic tissue, where they could evoke potentially life-threatening pathology [7].

The mesenteric lymph node (MLN) is the main draining lymph node in mouse enterocoelia which contains many types of immune cells [8]. MLN has been associated with initiation of immunological responses to bacterial translocation and inflammatory bowel diseases (IBDs) [9]. Moreover, it was reported that MLN CD4^+^ T lymphocytes could migrate to liver and contribute to nonalcoholic fatty liver disease [10]. Our previous study have found that *S. japonicum* infection could stimulate the responses of multiple immune cells, including Th cells, NK cells, NKT cells, and *γδ*T cells in the B6 mouse MLN [11, 12].

CD4^+^ Th cells could modulate the immune response by secreting many kinds of cytokines. According to the different cytokine production profiles, divide into different subtypes, such as Th1, Th2, Th9, and Th17 [13]. It was reported that CD4^+^ Th2 cell is the main effector T cell response to *S. japonicum* infection by producing produce IL-4, IL-5, and IL-13 [14]. IL-17-secreting Th17 cell was reported playing an important roles in *S. japonicum* infection inducing liver granuloma damage [4, 5]. Th9 cells could influence the progress of *S. japonicum* infection-induced liver damage, too [15]. IFN-*γ* and IL-4 were classic Th1 and Th2 cytokines, respectively. IFN-*γ* could mediate cellular immune response, including the activity of CD8^+^ cytotoxin T cell and macrophages. On the contrary, IL-4 is the most important cytokine in induced B cell activation and antibody production. IFN-*γ* and IL-4 were the most important cytokines secreting by Th cell which influence the progress *S. japonicum* infection-induced disease [16].

TLRs are the best characterized class of pattern recognition receptors (PRRs) that prevent pathogen invasion by recognizing pathogen-associated molecular patterns (PAMPs), which are highly conserved components derived from bacteria, viruses, fungi, and parasites [17]. Studies show that TLRs are the most important sensors to parasite components during *Schistosoma mansoni* infection [18, 19]. Although TLRs are predominantly expressed in innate immune cells, such as dendritic cells, macrophages, and natural killer (NK) cells [20]. TLRs have also been detected in T cells [21] and were found to be able to modulate the function of T lymphocytes [22]. For example, the report by Lee et al. indicated that TLR2 was constitutively expressed on *Listeria*-specific memory CD8^+^ T cells [23]. In addition, Caron et al. reported that effector memory T cells exhibit an enhanced response to TLR activation and are more sensitive to TLR-mediated activation than naive CD4^+^ T cells [24].

Among all the TLRs identified, TLR7 is an intracellular member of the innate immune receptor that recognizes intracellular single-stranded and double-stranded RNA [25]. It was reported that TLR7 was involved in the progress of autoimmune disease [26], graft-versus-host disease [27], and infectious diseases [28]. For example, TLR7 could be detected on different CD4^+^ and CD8^+^ T cell subpopulations from blood of hepatitis C virus infected patients by flow cytometry [29]. Resiquimod (R848), a TLR7 and TLR8 agonist, could not only induce immune response as an adjuvant [30], but also blocks virus replication by inducing the antiviral protein viperin [31]. To date, however, the exact role of the TLR7 in *S. japonicum* infection remains elusive. In this study, we utilized both *in vivo* TLR7 gene knockedout (KO) mice and *in vitro* schistosome worm (SWA)- and egg (SEA)-stimulated mesenteric lymphocytes to investigate the roles of TLR7 on T cells residue in the mesenteric lymph node (MLN) in the course of *S. japonicum* infection.

## 2. Materials and Methods

### 2.1. Ethics Statement

Animal experiments were performed in strict accordance with the Regulations for the Administration of Affairs Concerning Experimental Animals (1988.11.1). All protocols for animal use were approved to be appropriate and humane by the Institutional Animal Care and Use Committee of Guangzhou Medical University (2012-11).

### 2.2. Mice, Parasites, and Infection

Sixty female C57BL/6 mice, 6 to 8 weeks old, weighted 20-25 g, were purchased from Guangdong Medical Laboratory Animal Center (Guangzhou, China), and TLR7 KO mice were purchased from the Jackson Laboratory (B6.129S1-Tlr7^tm1Fl^v/J, strains: 008380). All mice were maintained in a specific pathogen-free microenvironment (SPF) at the Laboratory Animal Centre, Guangzhou Medical University. Mice were fed with standard diet, allowed ad libitum access to food and water and taken care of on a 12 h light-dark cycle. *S. japonicum* cercariae were shed from naturally infected *Oncomelania hupensis* snails, which were purchased from Jiangsu Institute of Parasitic Disease (Wuxi, China). There are 3 groups of mice in this study. 40 C57BL/6 mice were divided into normal and infected group randomly, twenty mice per group. 20 C57BL/6 mice in the infected group and 10 TLR7 KO mice (TLR7 KO group) were infected percutaneously with 40 ± 5 cercariae and sacrificed at 6 weeks after infection; 10 uninfectedTLR7 KO mice were served as control, too. The animal experiments were performed in strict accordance with the regulations for the Administration of Affairs Concerning Experimental Animals, and all efforts were made to minimize suffering. The bodies of the mice were frozen in -20°C and sent to the Laboratory Animal Centre of Guangzhou Medical University after the experiment.

### 2.3. Antibodies

FITC-conjugated anti-mouse CD8 (53-6.7), PerCP-cy5.5-conjugated anti-mouse CD4 (RM4-5), PE-conjugated anti-mouse CD25 (3C7), APC-conjugated anti-mouse CD69 (H1.2F3), APC-conjugated anti-mouse CD3 (145-2C11), APC-cy7-conjugated anti-mouse CD3 (145-2C11), PE-conjugated anti-mouse TLR4 (MTS510), APC-conjugated anti-mouse IFN-*γ* (XMG1.2), PE-conjugated anti-mouse IL-4 (11B11), and APC-conjugated anti-mouse TLR3 (11F8) were purchased from BD Pharmingen (San Diego, CA, USA). FITC-conjugated anti-mouse TLR2 (T2.5) and FITC-conjugated anti-mouse CD127 (ATR34) were purchased from Bio-Legend (San Diego, CA, USA). Purified anti-mouse CD3 (145-2C11) and anti-mouse CD28 (37.51) were purchased from BD Pharmingen (San Diego, CA, USA).

### 2.4. SEA and SWA Preparation

SEA and SWA were obtained from the Jiangsu Institute of Parasitic Diseases as previously described [32]. In brief, SEA and SWA were sterile filtered and the endotoxin was removed with polymyxin B agarose beads (Sigma-Aldrich). A Limulus amebocyte lysate assay kit (Lonza, Basel, Switzerland) was used to confirm the removal of the endotoxin from SEA and SWA.

### 2.5. Isolation of Lymphocytes

At 6 weeks after infection, the mice were sacrificed by cervical dislocation in laboratory, and the mesenteric lymph nodes (MLN) were harvested [11, 12]. A 100 *μ*m cell strainer (BD, CA, USA) was used for preparing the single cell suspensions. The isolated cells were washed twice in Hanks' balanced salt solution, stained by 0.4% trypan blue (Guangzhou chemical reagent factory), and counted under a microscope (the rate of ling cell >98%). The cells were resuspended and adjusted to 2 × 10^6^ cells/ml in complete RPMI-1640 medium supplemented with 10% heat-inactivated fetal calf serum, 100 U/ml penicillin, 100 *μ*g/ml streptomycin, 2 mm glutamine, and 50 *μ*m 2-mercaptoethanol.

### 2.6. Total RNA Isolation and Quantitative Real-Time PCR (qRT-PCR)

2 × 10^6^ cells from LN of both infected and normal groups were collected. Total RNA was isolated from the MLN cells of infected and normal mice using the TRIzol Reagent (Invitrogen Life Technologies, Carlsbad, CA, USA), following the manufacturer's instructions. The relative expression of each TLR mRNA was determined by real-time PCR using the ABI Prism 7500 Real-Time PCR System (Life Technologies) with SYBR ® Premix Ex Taq II (Tli RNaseH Plus) (Takara), according to the manufacturer's instructions. The cycle threshold (Ct) numbers were derived from the exponential phase of PCR amplification. The cDNAs were amplified under conditions of initial denaturation at 95°C for 10 minutes, followed by 40 cycles with denaturation at 95°C for 15 seconds, annealing at 60°C for 30 seconds, and extension at 72°C for 30 seconds. The levels of TLR2, TLR3, TLR4, and TLR7 transcripts were normalized to *β*-actin transcripts, using the relative quantity (RQ) = 2^−△Ct^ method.

The primers were synthesized from Invitrogen (Shanghai, China) as follows: for *β*-actin, 5-CCGTAAAGACCTCTATGCCAAC-3 (forward) and 5-GGGTGTAAAACGCAGCTCAGTA-3 (reverse); for TLR2, 5-AAGATGTCGTTCAAGGAGGTGCG-3 (forward) and 5-ATCCTCTGAGATTTGACGCTTTG-3 (reverse); for TLR3, 5-CCTCTTCATAATCAGCACCAG-3 (forward) and 5-CCAAGAATCCGATGCACTGA-3 (reverse); for TLR4, 5-ACCTGGAATGGGAGGACAATC-3 (forward) and 5-AGGTCCAAGTTGCCGTTTCT-3 (reverse); and for TLR7, 5-CCACATTCACTCTCTTCATTGG-3 (forward) and 5-GGTCAAGAACTTCCAGCCTG-3 (reverse).

### 2.7. ELISA Detection of Cytokines

Single cell suspensions from the normal, infected, and TLR7 KO group were prepared, respectively. Cells were plated in 96-well plates at 4 × 10^5^ cells/200 *μ*l medium per well and cultured for 72 h at 37°C with 5% CO_2_ in the presence or absence of anti-CD3 mAb (1 *μ*g/ml) plus PAMPs (PGN, 10 *μ*g/ml, Poly I:C 25 *μ*g/ml, LPS 1 *μ*g/ml, or R848 2 *μ*g/ml) or not. The supernatants were collected 72 h later and the released cytokines were measured using mouse ELISA kits for IFN-*γ* (R&D Systems Inc., Minneapolis, MN, USA) and IL-4 (BD Pharmingen, Franklin Lakes, NJ, USA). ELISAs were performed in accordance with the manufacturer's instructions. The optical density of each well was read at 450 nm using a microplate reader (Model ELX-800; BioTek Instruments Inc., Winooski, VT, USA).

### 2.8. Cell Surface and Intracellular Cytokine Staining (ICS)

For cell surface staining, single cell suspensions from the MLN of the normal group, infected group, and TLR KO group were washed twice in PBS contained 0.5% BSA and then stained for 30 min at 4°C in the dark with conjugated antibodies specific for the cell surface antigens CD3, CD4, CD8 CD25, CD69, TLR2, and TLR4. Cells were washed twice in PBS, fixed with 4% paraformaldehyde, and permeabilized overnight at 4°C in PBS buffer containing 0.1% saponin (Sigma), 0.1% BSA, and 0.05% NaN_3_. The cells were then stained for 30 min at 4°C in the dark with conjugated antibodies specific for TLR3 and TLR7. Stained cells were washed twice and detected by using flow cytometry (Cytoflex, Beckman Coulter, USA) and data were analyzed by the program CytExpert 1.1 (Beckman Coulter, USA).

For intracellular cytokine staining, single cell suspensions from the MLN of control mice and mice infected with *S. japonicum* were stimulated with TLR ligands (PGN 10 *μ*g/ml, Poly I:C 25 *μ*g/ml, LPS 1 *μ*g/ml, or R848 2 *μ*g/ml) plus 1 *μ*g/ml anti-CD3 for 5 h at 37°C under a 5% CO_2_ atmosphere. Brefeldin A (1 *μ*g/ml, Sigma) was added during the last 4 h of incubation. The cells were washed twice in PBS and stained for 30 min at 4°C in the dark with conjugated antibodies specific for the cell surface antigens CD3, CD4, and CD8. The cells were washed twice in PBS, fixed with 4% paraformaldehyde, and permeabilized overnight at 4°C in PBS buffer containing 0.1% saponin (Sigma), 0.1% BSA, and 0.05% NaN_3_. The cells were then stained for 30 min at 4°C in the dark with conjugated antibodies specific for the intracellular IFN-*γ* and IL-4. Stained cells were washed twice and detected by using flow cytometry (Cytoflex, Beckman Coulter, USA), and data were analyzed by the program CytExpert 1.1 (Beckman Coulter, USA).

### 2.9. Statistics

Data from each group were analysed using SPSS (v11.0). Statistical evaluation of the difference between means was performed by unpaired, two-tailed Student's *t*-tests; *P* < 0.05 was considered to be significant.

## 3. Results

### 3.1. Accumulation of CD3^+^ T Cells in Infected Mesenteric Lymph Nodes

**Figure 1:**
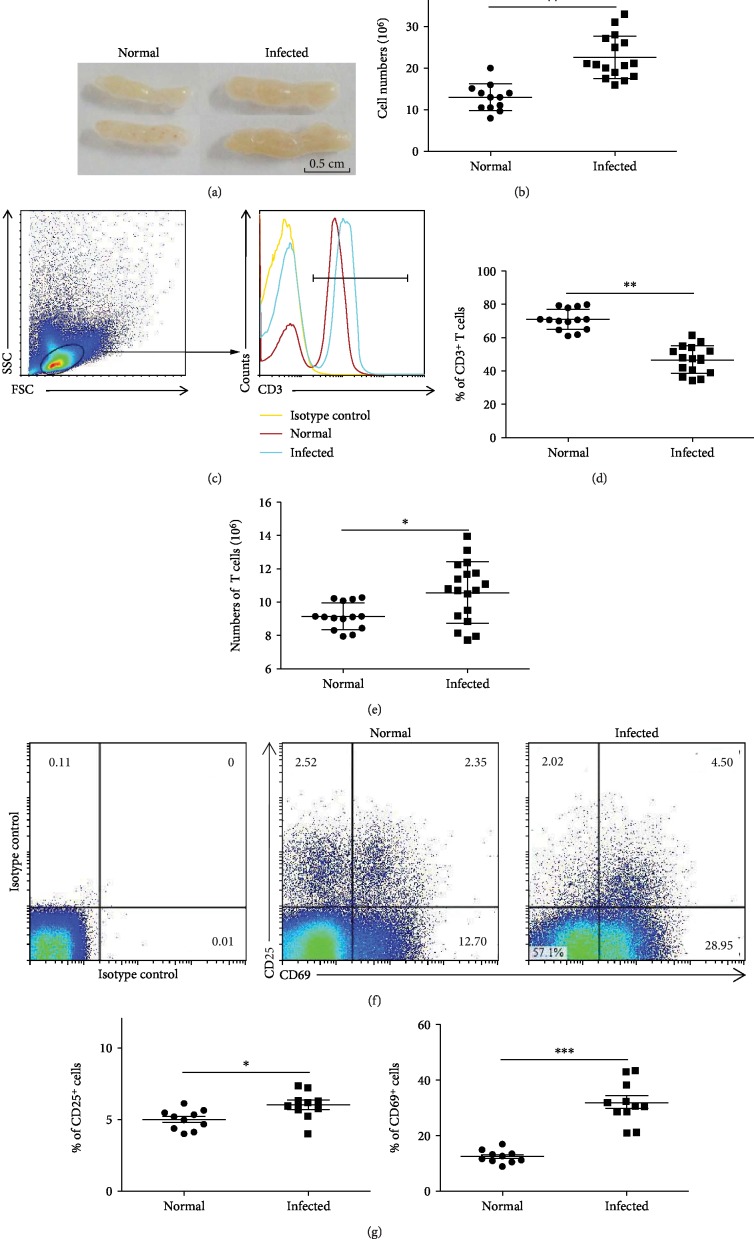
*Schistosoma japonicum* infection promotes CD3^+^ T cell accumulation in mesenteric lymph nodes. Female C57BL/6 mice were infected with 40 ± 5 S*. japonicum* cercariae per mouse. The mice were sacrificed 6 weeks after infection. The tissues and single cell suspensions were harvested. (a) Representative images of mesenteric lymph nodes. (b) Single mononuclear cell solutions were stained by trypan blue, and the absolute numbers were counted under a microscope (15/20). (c) Flow cytometric analysis of CD3 expression in MLN cells of normal and infected mice is shown. (d) Average percentages of CD3^+^ T cells were calculated from the FACS analysis (15/20). (e) The absolute number of CD3^+^ T cells evaluated by flow cytometry after staining with specific antibodies (15/20). (f) Flow cytometric analysis of CD25 and CD69 expression in MLN cells is shown. The numbers represent the expression of cells in each subset. (g) Average percentages of CD25 and CD69 expressions in the CD3^+^ T cells were calculated from FACS data. Data was from three independent experiments with 5 mice per group and shown as the mean ± SEM. ^∗^*P* < 0.05, ^∗∗^*P* < 0.01.

Six weeks after infection, the mice were sacrificed, and MLNs were harvested. Compared to the normal group, the infected MLN had significantly increased in size (Figure 1(a)). Single mononuclear cell solutions were prepared and stained by trypan blue; the living cells were counted. The average number of cells in nontreated MLN was (12.88 ± 3.26) × 10^6^. This number significantly increased to (22.54 ± 5.90) × 10^6^ after 6 weeks of infection (Figure 1(b), *P* < 0.01).

To investigate whether MLN T cells were involved in the host response to *S. japonicum* infection, mononuclear cells from normal or infected mouse MLN were stained by fluorescence-labeled anti-CD3 antibody and were detected by FACS (Figure 1(c)). As shown in Figure 1(d), the percentage of CD3^+^ T cells in infected mouse MLN was 46.76 ± 8.43%, which was lower compared to normal mice (70.9 ± 6.31%, *P* < 0.01). However, because of the number of MLN mononuclear cells in response to infection dramatically increased, the absolute number of MLN CD3^+^ T cells was obviously increased after infection (*P* < 0.05, Figure 1(e)).

CD25 and CD69 were classic markers for T cell activation [33]. To detect the degree of activation, the expression of CD25 and CD69 on CD3^+^ T cells was detected by the cell surface staining. As shown in Figure 1(f), the expression of CD69 on the CD3^+^ cells after infection (31.87 ± 9.58%) was significantly higher than normal mice (12.26 ± 2.54%, *P* < 0.05, Figure 1(g)). However, no significant change was found in the population of CD3^+^CD25^+^ T cells (*P* > 0.05, Figure 1(g)).

### 3.2. Expression of TLRs in S. Japonicum-Infected Mouse MLN

**Figure 2:**
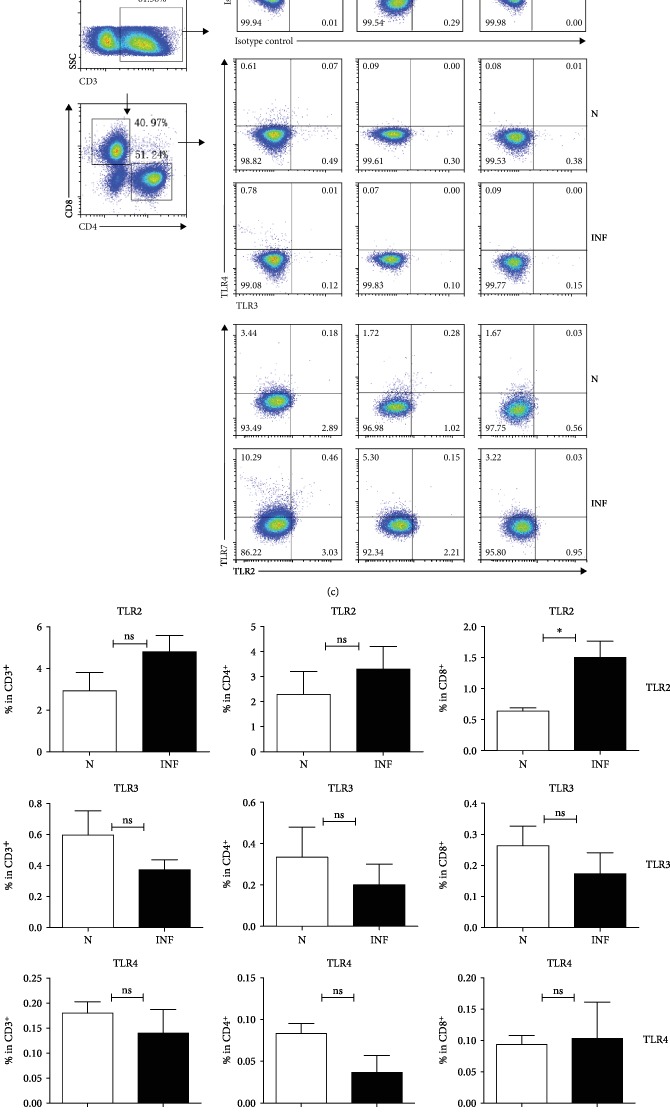
Expression of TLRs in the MLN of *S. japonicum-*infected mouse. Six weeks after *S. japonicum* infection, the mice were sacrificed. Single mononuclear cell suspensions from normal and infected mice were prepared as described in Materials and Methods. Total RNA was collected and purified, and cDNA was synthesized. (a, b) The relative mRNA expression of TLRs and *β*-actin genes was detected. Data was from three independent experiments with 5 mice per group and shown as the mean ± SEM. ^∗^*P* < 0.05, ^ns^*P* > 0.05. (c, d) Single cell suspensions of MLN cells were prepared, and the expression of TLR2, TLR3, TLR4, and TLR7 on CD4^+^ or CD8^+^ T cells was detected by flow cytometry after staining with specific antibodies. (c) The numbers represent the expression of cells in each subset. (d) Average percentages of TLR2, TLR3, TLR4, and TLR7 on CD4^+^ or CD8^+^ T cells were calculated from FACS data. Three independent experiments (5–6 mice per group) were performed, and one representative result is shown. ^∗^*P* < 0.05, ^ns^*P* > 0.05.

To explore the expression changes of TLRs in the *S. japonicum*-infected mouse *MLN*, we isolated MLN from both normal and infected mice and performed the qRT-PCR. The expression of TLR2, TLR3, TLR4, TLR7, and *β*-actin genes was detected as described in Materials and Methods. As shown in Figures 2(a) and 2(b), the amount of TLR7 mRNA in infected mice (2.040 ± 0.2062) was higher than that in nontreated mice (1.327 ± 0.1436, *P* < 0.05). Although there were also changes in the expression of TLR2, TLR3, and TLR4, the difference was not statistically significant (*P* > 0.05).

Moreover, the frequency of TLR2, TLR3, TLR4, and TLR7 on CD3^+^, CD4^+^, and CD8^+^ T cells was detected by flow cytometry after staining with specific antibodies as described in Materials and Methods. Results (Figures 2(c) and 2(d)) showed that the percentages of TLR7^+^ cells in the infected mice were higher than normal on CD3^+^, CD4^+^, and CD8^+^ T lymphocytes (CD3: 9.9 ± 0.86%*vs.*5.25 ± 0.91%; CD4: 6.62 ± 0.96%*vs.*3.21 ± 0.43; CD8: 3.12 ± 0.25%*vs.*1.65 ± 0.7%, *P* < 0.05). There was no significant difference on frequency of the rest of TLRs between the two groups, except TLR2 on CD8^+^ T lymphocytes (1.51 ± 0.26%*vs.*0.63 ± 0.18%, *P* < 0.05).

### 3.3. IFN-*γ* and IL-4 Induced by TLR Agonists

**Figure 3:**
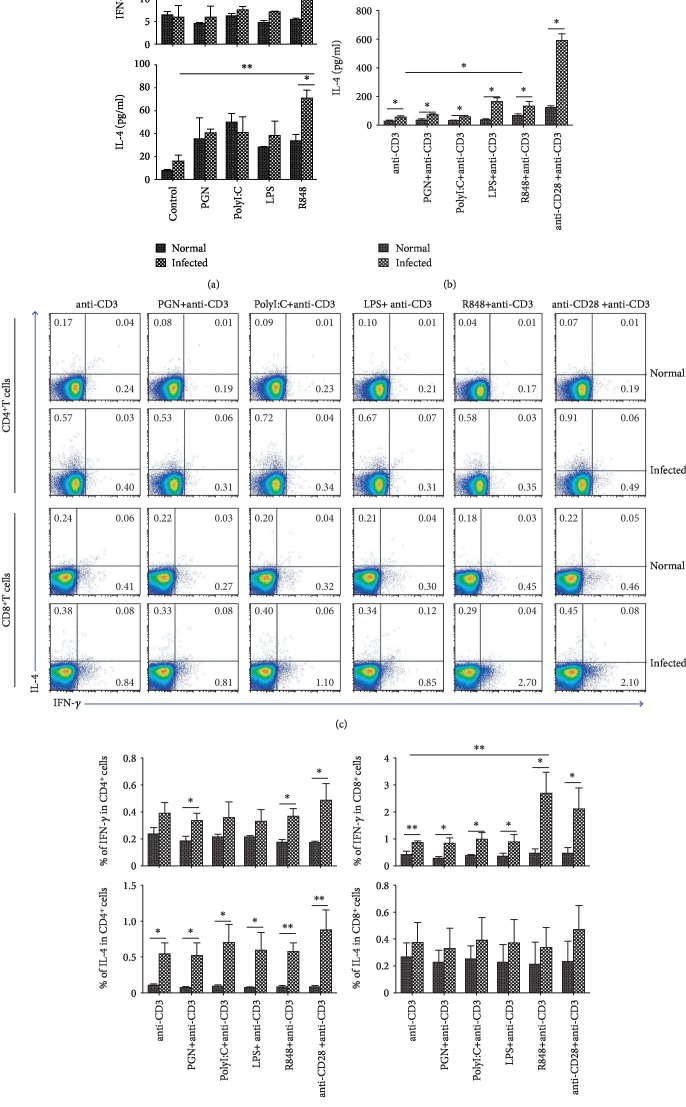
Role of TLR agonists in inducing IFN-*γ* and IL-4. Single mononuclear MLN cell suspensions of normal and infected mouse were prepared and cultured in vitro with PGN, PolyI:C, LPS, and R848, with or without anti-CD3 Ab. 72 h later, the concentration of IFN-*γ* (a) and IL-4 (b) in the supernatants of cultured cells was detected by ELISA. The MLN lymphocytes isolated from normal and infected mice were stimulated by PGN, Poly I:C, LPS, and R848, with anti-CD3 Ab. The expression of INF-*γ* and IL-4 on CD4^+^ or CD8^+^ T cells in normal and infected mouse was detected by flow cytometry. (c) The numbers represent the expression of cells in each subset. The average percentages of INF-*γ* and IL-4 in CD4^+^ (d) or CD8^+^ (e) T cells were calculated from the FACS analysis. Three independent experiments (5–6 mice per group) were performed, and one representative result is shown. ^∗^*P* < 0.05, ^∗∗^*P* < 0.01, ^ns^*P* > 0.05.

To explore the roles of TLRs in the function of T cells, the suspensions of single mononuclear cells from the MLN of normal and infected mice were cultured with PGN, Poly I:C, LPS, or R848, respectively, with or without anti-CD3 Ab, and the expression of cytokines was detected by ELISA. As shown in Figure 3(a), R848 showed a strong effect in promoting the production of IFN-*γ* and IL-4 from infected mouse MLN cells (IFN-*γ*: 19.59 ± 1.00 pg/ml*vs.*5.54 ± 0.21 pg/ml, IL-4: 70.39 ± 6.82 pg/ml*vs.*33.37 ± 5.52 pg/ml, *P* < 0.05). This effect was obvious in the presence of anti-CD3 Ab (IFN-*γ*: 826.31 ± 54.07 pg/ml*vs.*365.36 ± 52.31 pg/ml, IL-4: 132.02 ± 32.40 pg/ml*vs.*65.37 ± 11.71 pg/ml, *P* < 0.05, Figure 3(b). When the cells were stimulated by LPS, PGN, or Poly I:C alone, little IFN-*γ* and IL-4 were induced (Figure 3(a)). With the stimulation by the CD3 antibody, LPS could induce a higher level of both IFN-*γ* (167.55 ± 40.94 pg/ml) and IL-4 (165.34 ± 22.23 pg/ml) in infected mouse cells than in the normal control (76.50 ± 16.26 pg/ml; 34.73 ± 6.69 pg/ml, *P* < 0.05, Figure 3(b)). PGN or Poly I:C-stimulated T cells could induce IFN-*γ* in infected mouse MLN cells, compared to normal control (PGN: 37.38 ± 6.14 pg/ml*vs.*9.43 ± 0.06 pg/ml; Poly I:C: 32.98 ± 2.12 pg/ml*vs.*21.30 ± 3.09 pg/ml, *P* < 0.05, Figure 3(a)). The same trend was observed in IL-4 production (PGN: 68.43 ± 5.44 pg/ml*vs.*33.00 ± 9.90 pg/ml; Poly I:C: 57.72 ± 6.02 pg/ml*vs.*33.03 ± 0.73 pg/ml, *P* < 0.05, Figure 3(b)).

Furthermore, MLN lymphocytes isolated from normal and infected C57BL/6 mice were stimulated by anti-CD3 Ab plus TLR agonists for 5 hours, and the IFN-*γ* and IL-4 expression on CD4^+^ T cells or CD8^+^ T cells was detected by FACS (Figure 3(c)). As shown in Figure 3(d), the percentage of IL-4^+^CD4^+^ T cells and IFN-*γ*^+^CD8^+^ T cells in infected mice was higher than that in normal mice, significantly (*P* < 0.05). After infection, CD4^+^ T cells displayed an increased capacity in producing IFN-*γ* also. The expression of IL-4 in CD8^+^ T cells from the infected MLNs was slightly increased compared with the normal MLNs. However, the difference was not significant (*P* > 0.05, Figure 3(e)). When compared to cells stimulated by anti-CD3 Ab alone, R848 induced a significant increase in the percentage of IFN-*γ*^+^CD8^+^ T cells (Figure 3(e), *P* < 0.05).

### 3.4. Phenotypic and Functional Changes of CD4^+^ and CD8^+^ T Cells from MLN of S. Japonicum-Infected TLR7 KO Mice

**Figure 4:**
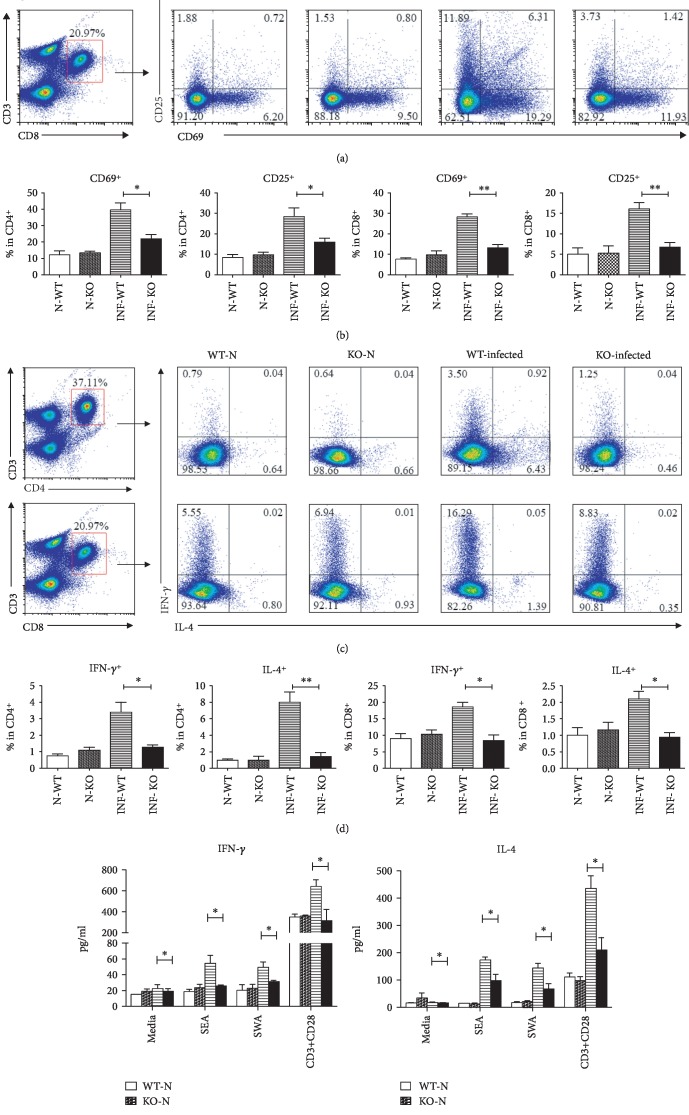
Change in activation and function of T lymphocytes of MLN after TLR7 knockout. T lymphocytes were isolated from wild-type normal (WT-N), TLR7 knockout normal (KO-N), wild-type infected (WT-infected), and TLR7 knockout infected (KO-infected) mice separately. The single cell solutions were prepared. (a, b) The expression of CD25 and CD69 on both CD4^+^ and CD8^+^ T cells was detected by the means of cell surface staining. (c, d) Cells were stimulated by PMA plus ionomycin; the expression of IFN-*γ* and IL-4 was detected by the means of intracellular cytokines staining as described in Materials and Methods. (e, f) Cells were cultured with plus SEA, SWA, and CD3, respectively, with CD28 for 72 hours. The concentration of IFN-*γ* and IL-4 was detected by the means of ELISA. Three independent experiments (5–6 mice per group) were performed, and one representative result is shown. ^∗^*P* < 0.05, ^∗∗^*P* < 0.01, ^ns^*P* > 0.05.

T lymphocytes were isolated from wild-type normal (WT-N), TLR7 knockout normal (TLR7-N), wild-type infected (WT-INF), and TLR7 knockout infected (TLR7-Inf) mice separately. The single cell solutions were prepared. The expression of CD25 and CD69 on both CD4^+^ and CD8^+^ T cells was detected by the means of cell surface staining as shown in Figure 4(a). The expressions of CD25 (CD4: WT-INF: 28.53 ± 4.08%, TLR7-INF: 15.85 ± 1.97%, *P* < 0.05; CD8: WT-INF: 16.07 ± 1.57%, TLR7-INF: 6.72 ± 1.17%, *P* < 0.01) and CD69 (CD4: WT-INF: 39.61 ± 4.33%, TLR7-INF: 21.94 ± 2.6%, *P* < 0.05; CD8: WT-INF: 28.2 ± 1.562%, TLR7-INF: 13.12 ± 1.74%, *P* < 0.01) from infected TLR7 KO mice were much lower than the WT-INF group on both CD4^+^ and CD8^+^ T lymphocytes (Figure 4(b)). It is suggesting the knockout of TLR7 influenced activation of T lymphocytes.

In the same time, cells were stimulated by PMA plus ionomycin; the expression of IFN-*γ* and IL-4 on both CD4^+^ and CD8^+^ T cells was detected by the means of intracellular cytokine staining as showed in Figure 4(c). Production of IFN-*γ* secreted by CD4 and CD8 T lymphocytes from wild-type infected mice were 3.4 ± 0.6% and 18.57 ± 1.45%, which were much higher than TLR7-INF (CD4: 1.27 ± 0.15%; CD8: 8.38 ± 1.72%, *P* < 0.05). Secretion of IL-4 of T lymphocytes from the WT-INF group (CD4: 8.01 ± 1.23%; CD8: 2.1 ± 0.23%) was also higher than TLR7-INF (CD4: 1.43 ± 0.48%; CD8: 0.94 ± 0.14%, *P* < 0.05) after infection (Figure 4(d)).

Moreover, lymphocytes from MLN were cultured with stimulation of SEA, SWA, or CD3, respectively, with CD28 for 72 hours. The concentration of IFN-*γ* (Figure 4(e)) and IL-4 (Figure 4(f)) in the supernatant of cultured cells was detected by ELISA. Results are shown in Figure 4(c); the production of IFN-*γ* (SEA: WT-INF: 61.78 ± 15.86 pg/ml, TLR7-INF: 21.8 ± 3.42 pg/ml, *P* < 0.05; SWA: WT-INF: 44.42 ± 3.33 pg/ml, TLR7-INF: 32.02 ± 2.35 pg/ml, *P* < 0.05) and IL-4 (SEA: WT-INF: 148.8 ± 19.41 pg/ml, TLR7-INF: 73.49 ± 14.27 pg/ml, *P* < 0.05; SWA: WT-INF: 98.92 ± 19.17 pg/ml, TLR7-INF: 42.09 ± 9.35 pg/ml, *P* < 0.05) from T lymphocytes of infected TLR7 knockout mice stimulated with SEA and SWA was less than the infected wild-type mice.

## 4. Discussion

In *S. japonicum* infection, T cells, stimulated mainly by the soluble adult worm antigens and soluble egg antigens of *S. japonicum*, were believed to play an important role in the infection-induced pathogenic immune response [11, 34]. In this study, the bigger size of MLN, which contained a greater number of CD3^+^ T cells, was found in the *S. japonicum*-infected B6 mouse. These results suggested that *S. japonicum* infection could induce strong immune response in intestinal tract. CD25 and CD69 were classic markers for T cell activation [33], though CD4^+^CD25^+^foxp3^+^ T cells were served as nature regulatory T cells (Treg) [35], and CD69 was seem to be a marker for tissue resident memory T cells (TRM) [36]. Higher percentages of CD25 and CD69-expressing CD3^+^ T were found in the infected mouse MLN. It further indicated that CD3^+^ T lymphocytes might be a component of the immune response during *S. japonicum* infection, as our previous study reported [12].

TLRs are the most well-described PRRs, which promote both innate defense mechanisms and adaptive immune responses to invasive pathogen infection [37]. Previous studies showed that TLR4 might be involved in the protection against *S. japonicum* infection [19], and the absence of TLR7 could influence the immune response against *S. japonicum* infection [19]. In this study, higher expression levels of TLR7 mRNA were found in infected MLN lymphocytes (*P* < 0.05), which suggested that TLR7 might involved in the infected-induced immune response in the lymph nodes. Recently, Applequist and Mac Leod et al. reported the detection of TLRs expression on T cells [21] and the functional roles of TLRs on the modulation of both CD4^+^ and CD8^+^ T lymphocytes [22]. Our FACS results showed that the expressions of TLR2 in CD4^+^ T cells and TLR7 in CD4^+^ and CD8^+^ T cells from infected mouse were higher than that in normal T cells (*P* < 0.05), especially the expression of TLR7 (*P* < 0.05). This finding suggested that TLR7 might have important effects directly on T cells in response to *S. japonicum* infection.

The binding of TLRs with their specific ligands could initiate a signaling cascade that results in the secretion of cytokines, which subsequently drives an inflammatory response and activates the adaptive immune system [38]. As shown in Figure 3, significant higher levels of IFN-*γ* and IL-4 could be induced by R848-stimulated lymphocytes from infected mice. It further confirmed that TLR7 played an important role in *S. japonicum* infection-induced immune response. It implied that many kinds of TLR7-expressing innate immune cells played an important role in this progress. Moreover, the ELISA and FACS results showed that TLR7 could help anti-CD3 antibody inducing IFN-*γ* releasing and promoting the percentage of IFN-*γ*^+^CD8^+^ T cells during *S. japonicum* infection. It implied that *S. japonicum* infection could induce a Th1 immune response and CTL activity through TLR7. Consistent with our results, the levels of Th1 cytokines, TNF-*α*, and INF-*γ* in the supernatant of cultured spleen cells from TLR7^−/−^ infected mice were found lower than those of WT mice [19]. Similarly, TLR7 was confirmed to promote Th1 polarization and may thus contribute to the pathogenesis of immune thrombocytopenia [39].

In the same time, we found that in the infected mice, the percentage of IFN-*γ* and IL-4 producing both CD4^+^ and CD8^+^ T cells induced by CD3 plus R848 was similar to that induced by CD3 plus CD28. However, significantly higher level of IFN-*γ* and lower level of IL-4 were induced by CD3 plus R848 in the supernatant of cultured cells from infected mice (*P* < 0.05). It meant that *S. japonicum* infection induce TLR7-expressing innate cells in the mesenteric lymph nodes apt to induce Th1 response.

Previous study, however, showed *S. japonicum* infection could induce a Th2-dominant immune response in the body [40]. To further evaluate the role of TLR7 in *S. japonicum* infection in the induction of T cell response in MLN, we performed further phenotypic and functional characterization of CD4^+^ and CD8^+^ T cells from both TLR7 KO mice and cultured lymphocytes. As showed in Figure 4, results indicated that decreased CD25, CD69, IFN-*γ*, and IL-4 expressed on CD4^+^ and CD8^+^ T cells from MLN of *S. japonicum-infected* TLR7 KO mice (*P* < 0.05). In the same time, ELISA results showed that both SEA- and SWA-specific IFN-*γ* and IL-4 decreased significantly in culture lymphocytes from MLN of *S. japonicum*-infected TLR7 KO mice (*P* < 0.05). Together, these findings imply that the effect of TLR7 might only play an early or limited effect on T cell responses in the course of *S. japonicum* infection.

In conclusion, this study indicated that *S. japonicum* infection could induce TLR7 expression in both CD4^+^ and CD8^+^ T cells of the MLN in C57BL/6 mice, and importantly, the alteration of TLR7 mediates T cell response in the early phase of infection. Further clinic investigations are warranted to define the roles of TLR7 in human host infection of *S. japonicum*.

## Data Availability

The datasets used in the current study are available from the corresponding authors on reasonable request.

## Abbreviations

TLR: Toll-like receptor
*S. japonicum*: *Schistosoma japonicum*
MLN: Mesenteric lymph node
RT-PCR: Real-time PCR
FACS: Fluorescence-activated cell sorter
KO: Knockedout
PGN: Peptidoglycan
Poly I:C: Polyinosinic-polycytidylic acid
LPS: Lipopolysaccharide
R848: Resiquimod
CD: Cluster of differentiation
ELISA: Enzyme-linked immunosorbent assay
ICS: Intracellular cytokine staining
Ab: Antibody
SEA: Soluble egg antigen
SWA: Soluble worm antigen.
